# Pathophysiology of Diet-Induced Acid Stress

**DOI:** 10.3390/ijms25042336

**Published:** 2024-02-16

**Authors:** Nimrit Goraya, Donald E. Wesson

**Affiliations:** 1 Department of Internal Medicine, Baylor Scott & White Health, Temple, TX 76508, USA; nimrit.goraya@bswhealth.org; 2 Department of Internal Medicine, Texas A&M Health Science Center College of Medicine, Temple, TX 76508, USA; 3 Department of Internal Medicine, Dell Medical School, The University of Texas at Austin, Dallas, TX 78712, USA

**Keywords:** acid base, bones, chronic kidney disease, diet, food, fruits, kidneys, muscles, vegetables

## Abstract

Diets can influence the body’s acid–base status because specific food components yield acids, bases, or neither when metabolized. Animal-sourced foods yield acids and plant-sourced food, particularly fruits and vegetables, generally yield bases when metabolized. Modern diets proportionately contain more animal-sourced than plant-sourced foods, are, thereby, generally net acid-producing, and so constitute an ongoing acid challenge. Acid accumulation severe enough to reduce serum bicarbonate concentration, i.e., manifesting as chronic metabolic acidosis, the most extreme end of the continuum of “acid stress”, harms bones and muscles and appears to enhance the progression of chronic kidney disease (CKD). Progressive acid accumulation that does not achieve the threshold amount necessary to cause chronic metabolic acidosis also appears to have deleterious effects. Specifically, identifiable acid retention without reduced serum bicarbonate concentration, which, in this review, we will call “covert acidosis”, appears to cause kidney injury and exacerbate CKD progression. Furthermore, the chronic engagement of mechanisms to mitigate the ongoing acid challenge of modern diets also appears to threaten health, including kidney health. This review describes the full continuum of “acid stress” to which modern diets contribute and the mechanisms by which acid stress challenges health. Ongoing research will develop clinically useful tools to identify stages of acid stress earlier than metabolic acidosis and determine if dietary acid reduction lowers or eliminates the threats to health that these diets appear to cause.

## 1. Introduction

Diet contributes to chronic disease and its outcomes [1], including cardiovascular disease (CVD) [2] and chronic kidney disease (CKD) [3]. A dietary factor associated with cardiovascular and kidney outcomes is its acid- or base-producing capacity. When metabolized, animal-sourced foods yield acids, and most plant-sourced foods, including fruits and vegetables (F & Vs), yield bases [4]. Typical modern diets are proportionately higher in animal-sourced foods than plant-sourced foods like F & Vs, making them net acid-producing [4]. Acid-producing diets eaten by individuals with CKD and severe reductions (<25% of normal) in their estimated glomerular filtration rate (eGFR) [5] can cause acid accumulation sufficient to reduce serum bicarbonate concentration ([HCO_3_^−^]) below the normal range of clinical laboratories, i.e., cause metabolic acidosis [6]. Metabolic acidosis in patients with CKD further increases the already increased CVD risk suffered by patients with CKD [7,8,9] and enhances CKD progression [10,11,12,13]. Acid-producing diets also contribute to identifiable acid accumulation that is not sufficient to reduce serum [HCO_3_^−^], i.e., not cause metabolic acidosis [14], a state variously called eubicarbonatemic acidosis [15,16], preclinical acidosis [17], or subclinical acidosis [18]. This state, which we will refer to as “covert acidosis”, is associated with decreased bone [19] and muscle [20] health, faster CKD progression [21,22], and increased CVD risk [23]. Acid-producing diets are associated with an increased CVD risk [24] and with increased incidence [25,26] and progression [27,28] of CKD in the general population, suggesting that these diets pose general threats to health. These data support the fact that acid-producing diets contribute to the full continuum of “acid stress” [14]. We will refer to these phases as late-, mid-, and early-phase acid stress in this review. We will also discuss emerging studies that are elucidating mechanisms related to how acid-producing diets cause acid stress and its associated adverse outcomes.

## 2. Dietary Contribution to Acid–Base Status

**Figure 1:**
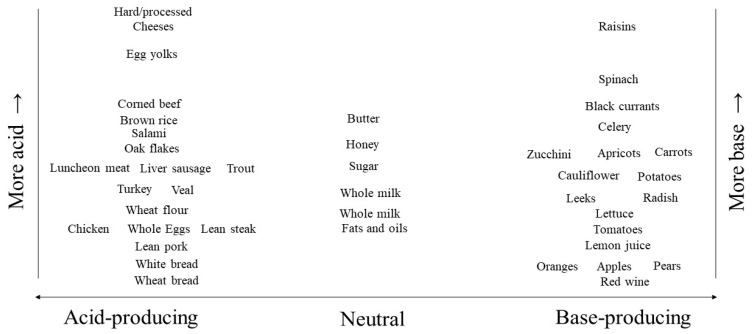
Graphic presentation of selected common foods that are generally acid-producing, neutral, or base-producing (horizontal orientation) and their comparable acid- or base-producing capacity (vertical orientation). The listed neutral foods are comparable with respect to their contribution to net endogenous acid production (NEAP), so their vertical orientation does not indicate ranking with respect to NEAP.

Both metabolic (called “fixed”) and respiratory (called “volatile”) acids challenge the acid–base status [29]. The accumulation of carbon dioxide (CO_2_) gas in bodily fluids causes *respiratory* acidosis and is typically due to decreased ventilation by the lungs [29]. On the other hand, metabolic acid accumulation, the topic of this discussion, causes the adverse consequences discussed above. Diets contribute metabolic acid through the metabolism of dietary proteins, phospholipids, and nucleic acids and incomplete carbohydrate oxidation [4]. This amounts to ~0.7–1.0 mmol/kg bw/day in healthy adults eating typical modern diets [30]. Proportionately more acid-producing animal-sourced foods than base-producing plant-sourced foods make these diets net acid-producing [4,30]. Animal-sourced foods have more protein/g and more sulfur-containing amino acids (e.g., methionine, cysteine) that metabolize to yield acids than plant-sourced foods [4,30]. Plant-sourced foods have more potassium and magnesium salts of organic anions like malate and citrate that metabolize to yield HCO_3_^−^ [4]. Figure 1 shows examples of acid-producing, base-producing, and neutral foods. Sodium chloride (NaCl) is common in processed foods that make up an increasing proportion of modern diets [31], and NaCl consumption is inversely related to serum [HCO_3_^−^] [32]. Unlike most processed foods, most fresh plant-sourced foods are very low in NaCl.

**Figure 2:**
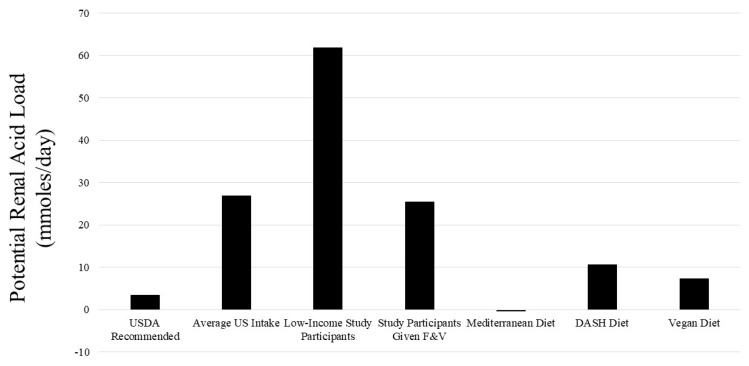
Graphic presentation of the potential renal acid load (PRAL), or net acid-producing capacity, of the indicated diets, calculated from their standard food components. The net acid-producing capacity of components of the United States Department of Agriculture (USDA)-recommended diet in this figure is considered the standard to which the remaining indicated diets are compared. F & Vs = fruits and vegetables; DASH = Dietary Approaches to Stop Hypertension. The data for diets of low-income study participants are from references [12,35,36,37]. The PRAL values for the remaining diets were calculated from typical food components of these diets and reported in reference [14].

Estimating a diet’s acid- or base-producing capacity can be achieved by calculating its dietary potential renal acid load (PRAL) [4]. This includes tabulating the type and quantity of foods in the diet and assigning their amount of acids (positive value) or bases (negative value) produced when metabolized [4]. This calculation does not include the estimated organic anion excretion (representing a potential base loss because retained organic anions can be metabolized to yield bases), which averages to about 40 mmol in adults and which, combined with PRAL, estimates urine net acid excretion (NAE) [4]. This comprehensive tabulation is labor-intensive, is largely carried out in research settings, and is not practically and routinely performed in clinical settings. The range of the net acid-producing capacity of common US diets is wide (Figure 2) but trends even higher in under-resourced communities [14]. The diets of residents in under-resourced communities trend toward higher PRALs because of a generally lower intake of plant-sourced foods, leading to a greater net metabolic acid production [14]. A lower intake of plant-sourced base-producing foods might mediate the greater risk of individuals from under-resourced disproportionately minoritized communities [33] to develop metabolic acidosis [34].

## 3. Overview of Acid–Base Balance

Cells and tissues seek to maintain “free” body fluid [H^+^] within a narrow, slightly alkaline range compared to pure H_2_O, which has [H^+^] = 100 nM or 100 × 10^−9^ M = 10^−7^ M = 10^−7^ moles/L. A solution’s pH is the negative log of its [H^+^] in moles/L, so pure H_2_O has a pH = 7.00. “Free” H^+^, as opposed to H^+^ bound to other moieties, appears to be the H^+^ component mediating its physiologic and pathophysiologic action, making its measurement clinically important. Nevertheless, most of the body’s H^+^ is indeed bound to other moieties (i.e., is “buffered”) not “free” in a solution. Buffered H^+^ appears to have less direct physiologic and/or pathophysiologic actions than free H^+^, but its degree of binding might signal downstream actions such as the level of kidney acidification [38]. Signal transduction pathways leading to increased H^+^ excretion induced by H^+^ accumulation can overlap pathways activating vascular endothelial cell inflammation [39], possibly mediating the increased CVD risk associated with increased dietary net H^+^ production. Consequently, accumulated H^+^ that is not “free” and so not measurable with conventional clinical tools might nevertheless be associated with and/or cause adverse clinical consequences. That being said, serum pH is routinely measured in clinical settings to help assess a patient’s acid–base status.

Multiple body systems help maintain serum [H^+^] at about 40 nM (pH = 7.40) despite dietary acid and/or base challenges and the intrinsic cellular metabolism. Individuals eating modern diets, typically net H^+^-producing [4], as previously discussed, who have severely decreased eGFRs due to CKD also have a decreased H^+^ excretory capacity. Others can have kidney tubule dysfunction that limits their H^+^ excretory capacity. As such, both groups can accumulate sufficient H^+^ to decrease serum [HCO_3_^−^], the metabolic component of the acid–base equilibrium, and develop *metabolic* acidosis. Others with less decreased GFRs, with intact tubule function, and eating acid-producing diets accumulate identifiable amounts of H^+^ that are not sufficient to reduce serum [HCO_3_^−^] [35,36,40,41] and yet appear to be sufficient to cause harm [19,20,21,22,23]. This setting is characterized by steady-state H^+^ retention without a decrease in serum [HCO_3_^−^] [42], a state to which we refer as covert acidosis. In addition, individuals without decreased GFRs might be able to avoid steady-state H^+^ retention in response to acid-producing diets [41], yet the chronic engagement of mechanisms to excrete H^+^ might lead to chronic kidney injury, as will be discussed later in this paper.

## 4. What Is a “Normal” Serum [HCO_3_^−^]?

A “normal” serum [HCO_3_^−^] varies widely among clinical laboratories, with low values as low as ≥18 mmol/L and high values as high as ≤36 mmol/L [43], lending uncertainty as to which serum [HCO_3_^−^] threshold indicates metabolic acidosis. Current clinical guidelines define metabolic acidosis as serum [HCO_3_^−^] < 22 mmol/L in the absence of respiratory alkalosis and recommend treatment for patients with CKD fitting this criterion [44]. By this measure, metabolic acidosis is prevalent in <1% of patients with CKD stage 1 [45], 1.3 to 7% of patients with CKD stage 2 (eGFR 60–89 mL/min/1.73 m^2^) [45,46], 13% with stage 3 (eGFR 30–59 mL/min/1.73 m^2^) [47], and 37% of patients with stage 4 (eGFR 15–29 mL/min/1.73 m^2^) [48]. Together, these data support that the most extreme form of acid stress, metabolic acidosis, is fortunately uncommon. As we will see later in this article, however, an identifiable acid accumulation that is not sufficient to reduce serum [HCO_3_^−^], that is, manifesting as metabolic acidosis, is also associated with adverse outcomes.

Despite the fact that sufficient acid accumulation to reduce serum [HCO_3_^−^], i.e., manifesting as metabolic acidosis, occurs in only a small minority of patients with CKD, epidemiologic and prospective interventional studies support that those with reduced eGFRs who eat acid-producing diets indeed present acid accumulation despite serum [HCO_3_^−^] levels within the normal ranges of clinical laboratories. Diets producing higher amounts of acids are directly associated with a higher anion gap in individuals with CKD; furthermore, the anion gap has been shown to be higher in those with eGFRs 30–59 mL/min/1.73 m^2^ than in those with eGFRs > 60 mL/min/1.73 m^2^, including individuals with serum [HCO_3_^−^] within the normal ranges [49]. In the same research, acid accumulation in those with reduced eGFRs and covert acidosis increased as the eGFRs further declined over time [49]. Furthermore, in another study, an increased amount of accumulated acid was directly associated with an increased plasma anion gap, despite the maintenance of serum [HCO_3_^−^] with the normal range [22]. Acid accumulation remained unchanged in participants receiving an oral alkali but increased in those not receiving it, showing the long-term benefit of chronic oral alkali therapy (five years) in reducing acid accumulation in covert acidosis cases [22] in addition to its shorter-term (30 days) benefits [40]. In addition, such a treatment in patients fitting these criteria reduced the urine indices of kidney injury in [21,36] and slowed the eGFR decline rate, supporting the notion that covert acidosis causes kidney injury and exacerbates eGFR decline. These data support the progressive course of acid stress, according to which individuals with reduced eGFRs who eat acid-producing diets can suffer harmful, kidney-toxic acid accumulation before it progresses towards reducing serum [HCO_3_^−^] below the normal range for clinical laboratories, the point at which current guidelines recommend treatment [44].

As will be discussed in more detail later, kidneys excrete H^+^ almost entirely bound to urine buffers and not “free” H^+^ [29]. The larger proportion of excreted H^+^ is bound to ammonium (NH_4_^+^), with the remainder being “titratable acidity”, mostly bound to phosphate.

## 5. Pathophysiology of Late-Phase H^+^ Stress (Metabolic Acidosis)

The extreme acid accumulation necessary to decrease serum [HCO_3_^−^] and thereby manifest as metabolic acidosis can be due to defective kidney tubule function limiting the kidney’s ability to excrete H^+^ despite a relatively well-preserved GFR. This can be due to dysfunction in the proximal or distal tubules. More commonly, the extreme acid accumulation of metabolic acidosis is mediated by a severe decrease (typically <25% of the baseline) in the GFR. This extreme level of body H^+^ accumulation is associated with injury to bones [50], muscles [51], the exacerbated progression of chronic kidney disease (CKD) [10,11,12,13], and an increased mortality [8], including an increased cardiovascular mortality [9].

### 5.1. Proximal Renal Tubule Acidosis

Individuals with proximal renal tubular acidosis (PRTA) have defective proximal tubule HCO_3_^−^ reabsorption with excess terminal nephron HCO_3_^−^ delivery that exceeds the capacity of the distal nephron to completely reabsorb the much higher load of delivered HCO_3_^−^. Because urine net acid excretion (NAE) = urine NH_4_^+^ + urine titratable acidity—urine HCO_3_^−^, excess urine HCO_3_^−^ excretion reduces urine NAE. These patients typically achieve a steady state of chronically low serum [HCO_3_^−^], at which the suboptimal proximal tubule more completely reabsorbs the lower load of filtered HCO_3_^−^ into the nephron (because of a lower serum [HCO_3_^−^]). This lower amount of HCO_3_^−^ delivery to the terminal nephron allows the functionally intact distal nephron to effectively excrete NH_4_^+^ and titratable acidity without excess urine HCO_3_^−^ excretion. This steady-state scenario allows the kidney to maintain a net acid balance, that is, match the dietary H^+^ intake with the H^+^ excretion. The steady-state price paid is that these patients have a steady-state H^+^ retention sufficient to reduce the serum [HCO_3_^−^], i.e., have chronic metabolic acidosis. Like nearly all patients with metabolic acidosis, they have a physiological response to the reduced serum [HCO_3_^−^], i.e., a decreased serum partial pressure of carbon dioxide gas (PCO_2_) [29].

The major clinical consequence of chronic metabolic acidosis in PRTA cases is inhibited bone growth in children [52]. Chronic metabolic acidosis is also associated with a low bone mineral content, as rickets in children, osteomalacia in adults, and nephrolithiasis in both [53]. The treatment of metabolic acidosis in PRTA requires large amounts of oral sodium bicarbonate (NaHCO_3_), typically 10–15 mEq/kg body wt daily [52,53], to maintain the serum [HCO_3_^−^] at a high enough level to avoid or ameliorate these adverse consequences. This treatment leads to large urine HCO_3_^−^ losses, forcing potassium and phosphate losses which themselves require replacement [52]. Although dietary acid reduction by limiting acid-producing foods and encouraging base-producing foods can be helpful, the large alkali requirements of patients with PRTA cannot be met with these dietary strategies alone.

### 5.2. Distal Renal Tubule Acidosis

Individuals with distal renal tubular acidosis (DRTA) have intact proximal tubular function and, therefore, do not deliver large HCO_3_^−^ loads to the terminal nephron like those individuals with PRTA. In contrast to PRTA, patients with DRTA have defective distal nephron acidification such that they have a lower excretion of NH_4_^+^ and/or titratable acidity [54,55]. Consequently, patients with DRTA are typically unable to completely excrete the high dietary H^+^ load typical of modern diets, so they present enough steady-state net H^+^ retention to reduce their serum [HCO_3_^−^] without treatment [55]. The retained H^+^ lowers the serum [HCO_3_^−^] (with a physiological PCO_2_ decrease), so these patients have chronic metabolic acidosis. The net acid retention causes bone diseases [53] and nephrolithiasis [56].

Because patients with DRTA have intact proximal tubular function, they do not have the large urine HCO_3_^−^ losses of PRTA, so they do not have the large alkali requirements of patients with PRTA. Instead, patients with DRTA require sufficient alkali to treat the described net H^+^ retention, which is much less than the amount required by patients with PRTA. Because the acid production capacity of modern society diets is typically ~1.0 mEq/kg body daily, most recommendations suggest that patients with DRTA be prescribed 1.0–1.5 mEq/kg body weight/day alkali equivalent daily, typically as NaHCO_3_ [55,57]. Oral alkali correction of metabolic acidosis due to DRTA in children has been shown to improve bone growth, mineral density, and histopathology [57,58]. Although there are no published studies describing DRTA treatment exclusively by limiting H^+^-producing or adding base-producing food constituents, the comparatively lower alkali requirements of patients with DRTA suggest that dietary strategies might be used at least as an adjunctive treatment to sodium (Na^+^)- or potassium (K^+^)-based alkali.

### 5.3. Chronic Metabolic Acidosis in Chronic Kidney Disease

Unlike the primary tubule dysfunction that mediates the reduced H^+^ excretion in RTAs, the reduced H^+^ excretion in so-called uremic acidosis, the most common cause of metabolic acidosis in CKD, is mediated by a severe reduction in filtration capacity, evidenced by severe reductions in the GFR [29]. In addition, these patients have reduced excretion of NH_4_^+^, the main urine buffer which binds H^+^, as discussed above. These two mechanisms combine to limit urine H^+^ to levels below those produced from a diet and the intrinsic metabolism, leading to an ongoing and increasing net H^+^ retention. When the level of H^+^ retention reduces the serum [HCO_3_^−^] below the normal range for clinical laboratories, these patients transition to metabolic acidosis.

The 2013 KDIGO guidelines [44] are the most structured recommendations for the treatment of chronic metabolic acidosis, but it is not clear whether these recommendations should be applied unmodified to other etiologies of chronic metabolic acidosis. These recommendations read as follows: “We suggest that in people with CKD and serum [HCO_3_^−^] < 22 mmol/L treatment with oral bicarbonate supplementation be given to maintain serum bicarbonate within the normal range, unless contraindicated”. The authors comment that the indicated serum [HCO_3_^−^] below which to treat patients has not been rigorously determined with large-scale studies but reflects the opinions and experience of the authors. The recommended doses range from 0.5 to 1.0 mEq HCO_3_^−^ or its equivalent per kilogram of lean body weight per day. This is similar in amount to that recommended for DRTA, because the concern with chronic metabolic acidosis in CKD is the failure to completely excrete the metabolically produced H^+^, mostly derived from dietary intake, with variable amounts contributed by the intrinsic metabolism. The treatment goal, as stated in KDIGO, is to maintain the serum [HCO_3_^−^] in the normal range. The guidelines recommend Na^+^-based alkali therapy such as Na^+^ citrate or NaHCO_3_, as tolerated by the patient. With evolving patient studies supporting the benefits of the alkali treatment of covert acidosis [21,35,36], updated guidelines recommend maintaining serum [HCO_3_^−^] levels at 24–26 mmol/L for patients with CKD and metabolic acidosis [59].

## 6. Pathophysiology of Mid-Phase H^+^ Stress (Covert Acidosis)

**Figure 3:**
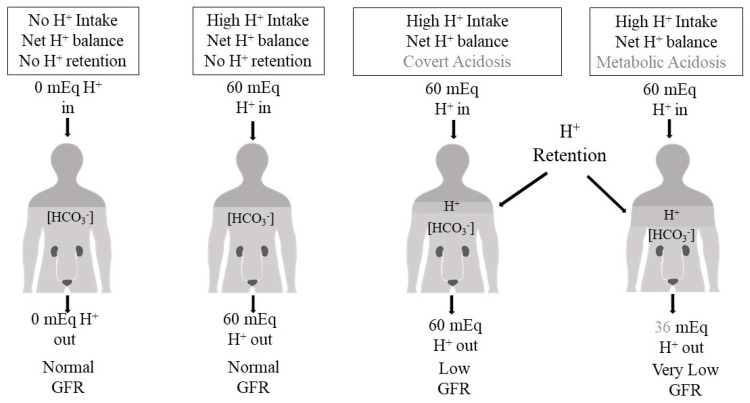
Conceptual representation of four phases of the continuum of acid stress. From left: individuals with a normal estimated glomerular filtration rate (eGFR, ≥90 mL/min/1.73 m^2^) and eating a no-acid (H^+^) diet, those with a normal eGFR and eating a typical H^+^-producing modern diet, those with a moderately reduced eGFR (30–89 mL/min/1.73 m^2^) and covert acidosis, and those with a severely reduced eGFR (<30 mL/min/1.73 m^2^) and metabolic acidosis. The light gray at the bottom of each figure represents the serum bicarbonate concentration ([HCO_3_^−^]), and its height indicates its level. The slightly darker gray above it represents acid (H^+^) retention acid, and the vertical height indicates its amount. The 60 mEq dietary H^+^ intake is the average intake of the study participants from under-resourced communities in references [12,35,36,41]. The amount of acid excretion for the right-most figure with metabolic acidosis is estimated using data from https://www.cabidigitallibrary.org/doi/full/10.5555/19731405301 (accessed on 13 February 2024). GFR = glomerular filtration rate.

Despite a steady-state H^+^ accumulation not sufficient to increase the serum [H^+^] (= decreased pH) and/or decrease the serum [HCO_3_^−^], the body’s intrinsic mechanisms to mitigate the accumulated H^+^ can cause injury. Even if the ongoing ingestion of H^+^-producing diets is not associated with an identifiable H^+^ accumulation, the chronic engagement of H^+^ excretory mechanisms promoted by H^+^-producing diets can also cause injury. Figure 3 represents a construct of the continuum of H^+^ stress that is supported by the experimental data to be discussed in this section.

## 7. Steady-State H^+^ Accumulation without Decreased Serum [HCO_3_^−^], i.e., Covert Acidosis

### 7.1. How Might Steady-State H^+^ Accumulation Occur?

**Figure 4:**
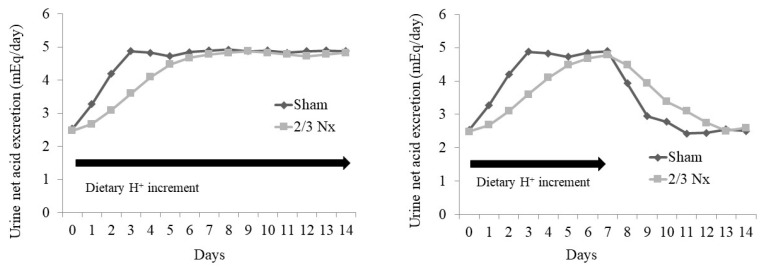
Illustration of data from reference #61 showing how acid retention occurred in rats with two-thirds of their nephron mass surgically removed (2/3 Nx) compared to sham-operated animals (Sham). The left panel shows the urine net acid excretion (UNAE) levels in the two animal groups over 14 days of an increment in dietary acid. The figure shows that, although both groups eventually reached steady-state UNAE which matched the ingested acid with the UNAE, the 2/3 Nx animals took longer to do so. The 2/3 Nx animals accumulated more acid in their bodies than the sham group during the longer time that it had taken them to achieve steady-state UNAE, as described in reference #61. The right panel depicts the course of UNAE in the two groups after a dietary acid increment that was discontinued after seven days. The figure shows that both groups eventually attained their baseline UNAE before the increment in dietary acid, but the 2/3 Nx animals took longer to do so.

Animal studies suggest mechanisms by which steady-state H^+^ accumulation might occur with minimal increases in the serum [H^+^] (decreased pH) or minimal decreases in the serum [HCO_3_^−^]. In a study, animals with CKD caused by the surgical removal of two-thirds of their nephron mass (2/3 Nx) had H^+^ retention detected via microdialysis and yet had serum acid–base parameters not different from the shams, despite eating the same H^+^-producing diet [60,61]. Both the 2/3 Nx group and the sham group achieved net H^+^ balance (i.e., dietary H^+^ intake = urine H^+^ excretion) when switched from a base-producing to an H^+^-producing diet, but the 2/3 Nx group took longer to do so [61] (Figure 4). Progressive H^+^ retention occurred during the longer time taken by the 2/3 Nx group to achieve net H^+^ balance, and this H^+^ retention was sustained while the animals ate the H^+^-producing diet; a baseline lower H^+^ retention recurred only when the animals returned to the base-producing diet [61], and the 2/3 Nx group took longer to return to their baseline urine net acid excretion (Figure 4). Similarly, the individuals without a known kidney disease given an increment in their dietary H^+^ intake cumulatively excreted less H^+^ than they ingested [62], which is consistent with sustained H^+^ accumulation in response to dietary H^+^ intake in humans.

### 7.2. Sequestration of Retained H^+^ into Non-Serum Fluid Compartments

Most (60–75%) H^+^ administered into the serum is sequestered intracellularly [63] and buffered [64]. Sequestering H^+^ from the serum into cells yields the benefit of reduced H^+^ exposure to tissues that interface with the serum. A detriment of this sequestering is that increased intracellular acidity releases iron bound to intracellular proteins, thereby forming hydroxyl radicals which cause tissue-damaging oxidative stress [65].

### 7.3. Buffering of Retained H^+^ by HCO_3_^−^

Adding H^+^ to body fluids containing HCO_3_^−^ leads to the following:

H^+^ + HCO_3_^−^ → H_2_CO_3_ → H_2_O + CO_2_ ↑ (lungs excrete CO_2_ gas from the body)

This leads to H^+^ removal from serum as CO_2_ gas that would yield H^+^ were it to accumulate (reversal of the above equation), thereby minimizing the increase in body fluid [H^+^]. Reduced serum [HCO_3_^−^] limits the body’s capacity to buffer further H^+^ accumulation. The restoration of the body’s HCO_3_^−^-mediated buffering capacity requires the kidneys to regenerate new HCO_3_^−^ through net H^+^ excretion, with the benefits and detriments which will be discussed under the subsequent section describing the effects of the ingestion of H^+^-producing diets.

### 7.4. Buffering of H^+^ by Non-HCO_3_ Buffers

A sustained dietary H^+^ increment in animals has been shown to cause an increment in their steady-state H^+^ accumulation, increasing the H^+^ titration of extracellular non-HCO_3_ buffers [38]. Bone calcium carbonate and dibasic phosphate chronically buffer dietary H^+^ that progressively reduces bone mineral content in patients [50]; released dibasic phosphate can contribute to titratable acidity for urine H^+^ excretion. Indeed, signs of metabolic acidosis-related disturbances of the mineral metabolism in CKD appear with declining eGFRs even before metabolic acidosis manifests itself via serum acid–base parameters [66], consistent with the adverse effects of early-phase H^+^ stress. Bone H^+^ buffering serves the short-term benefit of mitigating injury from accumulating H^+^ but incurs the longer-term detriment of reduced bone mineral content, with the related decreased bone integrity and increased risk for fracture [50].

### 7.5. H^+^ Neutralization by Endogenous Organic Acids

Citrate is the most abundant organic base-equivalent in urine, and its level of proximal tubule reabsorption determines its urine excretion rate [67]. Secreted H^+^ into the proximal tubule lumen partially titrates filtered citrate^3−^ to Hcitrate^2−^, the latter being the preferred substrate for the apical Na^+^ dicarboxylate cotransporter NaDC-1 [68]. Cytoplasmic ATP citrate lyase metabolizes reabsorbed citrate to oxaloacetate and acetyl-CoA or transports it into the mitochondria, where it enters the tricarboxylic acid cycle [69]. The metabolism of retained citrate yields HCO_3_^−^, constituting base gain, but its excretion constitutes base loss [70]. Individuals with reduced eGFRs eating highly H^+^-producing diets and presenting covert acidosis had lower urine citrate excretion rates [35,36,41], as did those eating highly H^+^-producing diets but without covert acidosis [16,71]. Reduced citrate stores, due to their consumption by accumulating H^+^, increase the risk for kidney stones [72].

### 7.6. Potential Mechanisms for Organ Injury Associated with Covert Acidosis

**Adverse effects on bone health.** Animals with covert acidosis have greater urine excretion of deoxy-pyridinoline, a biomarker of bone matrix injury [61]. Chronic acid buffering by bones likely contributes to the increased bone turnover associated with this phase of acid stress [26]. Covert acidosis that occurs because of the combination of progressive GFR decrease and acid-producing modern diets has been proposed to contribute to the decreased bone mass observed in aging [73].

**Adverse effects on skeletal muscle health.** Most NAE occurs through ammoniagenesis. In this process, kidneys metabolize the amino acid glutamine, derived, in part, from the breakdown of skeletal muscle protein, to form ammonia (NH_3_) and α-ketoglutarate [29]. Ammonia binds H^+^ to yield ammonium (NH_4_^+^) (NH_3_ + H^+^ → NH_4_^+^), which facilitates acid removal from the body through urine NH_4_^+^ excretion. The α-ketoglutarate can be metabolized to HCO_3_^−^ to replace the HCO_3_^−^ that was lost through titration by the accumulated acid. In this way, ammoniagenesis yields the benefit of acid removal from the body with the replacement of the HCO_3_^−^ that was lost through acid titration, thereby completing NAE [29]. When sustained, as in animals, with covert acidosis, chronic ammoniagenesis likely contributes to the loss of muscle mass associated with covert acidosis in patients [20]. In addition, the treatment of patients with CKD and serum [HCO_3_^−^] 20–24 mmol/L (mean = 23 mmol/L, suggesting that most patients have serum [HCO_3_^−^] > 22 mmol/L) with oral NaHCO_3_ has been associated with increased skeletal muscle mass [74]. These data suggest that the chronic activation of kidney NAE mechanisms in patients with covert acidosis threatens skeletal muscle mass.

**Adverse effects on kidney health.** Animal models of CKD with covert acidosis have high kidney levels of angiotensin II, aldosterone, and endothelin-1 (ET-1), each of which stimulates kidney distal nephron acidification to increase NAE [75,76]. This short-term benefit of increased NAE is accompanied by the longer-term detriment of kidney injury with a progressive GFR decline when the kidney levels of these agents remain chronically high [75,76]. Chronic oral NaHCO_3_ in study participants with CKD and covert acidosis has been shown to reduce accumulated acid, urine excretion of aldosterone and ET-1, and urine indices of kidney injury, consistent with kidney injury caused by covert acidosis [21,36]. As mentioned, ammonium (NH_4_^+^) constitutes most urine NAE [29]. Animal studies show and patient studies support the idea that acid-producing diets stimulate kidney NH_4_^+^ production that enhances a complement [37,77]. In turn, this complement mediates kidney interstitial inflammation with progressive kidney function decline [37].

## 8. Potential Strategies for Detecting Covert Acidosis

As noted, steady-state covert acidosis more likely occurs in individuals eating acid-producing diets with CKD and reduced eGFRs. These patients appear to be suitable candidates for an investigation trying to determine whether they have covert acidosis. Low urine NH_4_^+^ excretion in patients with CKD and reduced eGFRs, possibly reflecting suboptimal urine NAE, has been associated with adverse kidney outcomes [77,78] and might identify individuals at risk for covert acidosis. In other studies, low urine citrate excretion indicated underlying covert acidosis in patients with CKD [22,35,41], their response to treatment with either mineral alkali [22] or base-producing food components 35, and changes in acid accumulation when followed over time [22]. Further studies will determine clinically useful indicators to identify individuals with covert acidosis.

## 9. Increased Dietary H^+^ with Little-to-No Steady-State H^+^ Retention (Early-Phase Acid Stress)

### 9.1. Effects of an Increment in Dietary H^+^ on Overall Body H^+^: Normal vs. Reduced GFR

Animals with a normal compared to a reduced GFR, both eating an H^+^-producing diet, were shown to attain net H^+^ balance faster with less H^+^ retention, even with similar baseline serum acid–base parameters [61]. After one week of a higher dietary H^+^ intake, both groups had increases in their urine indicators of bone and kidney injury, which returned to the baseline with the restoration of the base-producing diet [61]. These data support the idea that animals with normal compared to reduced GFRs more rapidly achieve net H^+^ balance in response to an increment in dietary H^+^, with less steady-state H^+^ retention. Nevertheless, even animals with normal baseline GFRs appear to suffer reversible bone and kidney injury in response to H^+^-producing diets [61]. Whether these insights apply to individuals with normal compared to reduced GFRs awaits further study.

### 9.2. Kidney Mechanisms Mediating the Excretion of Dietary H^+^ That Might Have Adverse Consequences

As indicated earlier, kidneys must accomplish the net excretion of accumulated metabolic H^+^ to restore the HCO_3_^−^-mediated buffering capacity that had been reduced by the accumulated metabolic H^+^. Kidneys accomplish net H^+^ excretion in ways that serve the short-term benefit of H^+^ removal but risk the long-term risk of injury to bones, muscles, and kidneys if the increment in dietary H^+^ is sustained. The kidney accomplishes net H^+^ excretion by means of two major mechanisms:

**Upregulation of kidney tubule H^+^ transporters.** Kidney tubule transporters secrete H^+^ into the tubule lumen that binds to urine buffers, mostly ammonium (NH_3_ + H^+^ → NH_4_^+^) and titratable acidity (PO_4_^=−^ + H^+^ → HPO_4_^=^ and HPO_4_^=^ + H^+^ → H_2_PO_4_^−^). These H^+^ transporters can be upregulated in response to dietary H^+^ with mineral H^+^ [79] or H^+^-producing dietary protein [80] by increased kidney levels of angiotensin II [76], aldosterone [81], and endothelin-1 (ET-1) [79,80] in experimental animals. The upregulation of angiotensin II [82] and aldosterone [83] is associated with reduced bone mass in experimental animals and patients, respectively. The upregulation of angiotensin II [84] and aldosterone [85] is associated with increased skeletal muscle atrophy and increased skeletal muscle apoptosis, respectively, in experimental animals. Higher kidney levels in each of these three substances have been shown to contribute to the short-term physiologic benefit of enhanced kidney acidification in experimental animals in response to this H^+^ challenge [80,81], but the long-term pathophysiologic detriment of increased kidney interstitial fibrosis [86,87], a component of progressive nephropathy [88], has also been observed. These data support the notion that dietary H^+^-induced decreases in the body’s HCO_3_^−^ leads to the upregulation of agents that decrease kidney function when engaged chronically.

Patients with modest eGFR reductions (CKD stage 2, eGFR 60–89 mL/min/1.73 m^2^) who eat highly H^+^-producing diets and have identifiable H^+^ retention can have increased urine excretion of ET-1 and aldosterone [40], consistent with increased kidney levels which help increase urine net H^+^ excretion but can contribute to a progressive GFR decline in animals, as discussed above. Treating such patients with oral NaHCO_3_ [40] or supplemental base-producing fruits and vegetables (F & V) [35] for 30 days has been shown to reduce H^+^ retention. Oral NaHCO_3_ for 30 days has been associated with decreased urine excretion of ET-1 and aldosterone [40] and, after five years, with a slower eGFR decline and reduced urine ET-1 excretion [21].

**Increased excretion of urine buffers.** Secreted H^+^ into the kidney tubule lumen quickly establishes an unfavorable concentration gradient for further luminal H^+^ secretion without luminal buffers to accept the secreted H^+^ and thereby limit “free” luminal H^+^. Among urine buffers, ammonium (NH_4_^+^) increases most in response to dietary H^+^ [89] and forms after the metabolism of the amino acid glutamine, which also yields α-ketoglutarate. Urine H^+^ excretion as NH_4_^+^, along with the metabolism of α-ketoglutarate to yield HCO_3_^−^, constitutes net H^+^ excretion with HCO_3_^−^ regeneration to replace that titrated by the accumulated H^+^.

Skeletal muscle protein is the major source of glutamine for NH_4_^+^ excretion in response to H^+^ challenges [90]. Oral mineral H^+^ in healthy volunteers was associated with stimulated protein degradation in skeletal muscle [91], highly H^+^-producing diets were associated with decreased skeletal muscle mass [92], and the treatment of chronic metabolic acidosis with mineral alkali was associated with increased skeletal muscle mass [74]. These data suggest that the kidneys’ net H^+^ excretion mechanisms in response to chronic H^+^ challenges threaten patient skeletal muscle mass.

Titratable acidity, mostly as dibasic phosphate, is the other major urine buffer accepting secreted H^+^. Its excretion increases less than that of NH_4_^+^ in response to a dietary H^+^ challenge [90]. Although bone is a source of dibasic phosphate which contributes to increased titratable acidity in response to dietary H^+^ [50], whether chronic highly H^+^-producing diets reduce bone mass remains unsettled [93].

## 10. How Clinicians Might Be Alerted to Patients at Risk for Early-Phase H^+^ Stress

Tests used in research settings to identify covert acidosis in patients with early-stage CKD [42] have yet to find utility in clinical settings. Because such patients have low urine citrate excretion [22,35,41], ongoing studies will determine whether this parameter has clinical utility in identifying this early-phase of H^+^ stress in patients with CKD and modestly reduced eGFRs but without metabolic acidosis according to their serum acid–base parameters. Although tabulating the types and amounts of foods eaten and calculating their acid- or base-producing potential [4] can reveal a high dietary H^+^, such efforts are not conducive to clinical settings. Because a high dietary H^+^ is associated with increased urine NH_4_^+^ excretion [41], studies are ongoing to determine whether this parameter has utility in identifying this even earlier phase of H^+^ stress. In the meantime, patients with CKD of stages 2 (eGFR 60–89 mL/min/1.73 m^2^) and 3 (eGFR 30–59 mL/min/1.73 m^2^) who are eating diets high in H^+^ appear to be at risk [41].

## 11. Potential Treatment for Early-Phase H^+^ Stress: Dietary H^+^ Reduction

Because most modern diets are H^+^-producing, dietary H^+^ reduction can be achieved by (1) limiting the intake of H^+^-producing diet components like animal-sourced foods; (2) adding base-producing plant-sourced foods like fruits and vegetables (F & V); and/or (3) adding Na^+^-based alkali (avoiding K^+^-based alkalis for patients with very low GFRs because of their reduced K^+^-excreting capacity) like NaHCO_3_ or sodium citrate.

Recent drug technologies have led to the development of oral, non-absorbable polymers that selectively bind gastric hydrochloric acid (HC1) and thereby remove body acid with feces [94].

### 11.1. Removing/Limiting H^+^-Producing Food Components

In a study, participants from under-resourced communities with CKD but no metabolic acidosis were given base-producing fruits and vegetables (F & Vs) free of charge and substituted these foods for the most expensive foods they had previously been eating, i.e., mostly processed meats. This strategy led to a decreased dietary H^+^ intake, reduced urine net acid excretion, and decreased urine indices of kidney injury [36]. This study supports the notion that dietary H^+^ reduction ameliorates kidney injury.

### 11.2. Adding Base-Producing Food Components

Adding base-producing foods like F & Vs can achieve dietary H^+^ reduction even without reducing the intake of H^+^-producing foods. The H^+^ content of many foods has been published [4] and can guide the recommendation of base-producing foods for patients with CKD. Adding 2–4 cups/day of F & Vs to diets of study participants with CKD, reduced eGFRs, and metabolic acidosis slowed CKD progression in [12]. The participants had eGFR > 30 mL/min/1.73 m^2^ and were identified as being at very low risk for hyperkalemia in response to the potassium load that accompanies F & Vs. Accordingly, clinicians must use caution when prescribing F & Vs to patients with CKD, particularly those with very low eGFRs, as a measure of kidney protection.

Diabetes and hypertension cause almost two-thirds of CKD in the US [5], and healthy diets including high proportions of F & Vs are recommended as the first-line therapy for each of these diseases [95,96]. Although an increased F & Vs intake helps to control both diabetes and hypertension, it is under-used in the management of both [95,96].

### 11.3. Na^+^-Based Alkali Therapies

Sodium bicarbonate (NaHCO_3_) is the typical oral mineral alkali used to reduce dietary H^+^ because it is effective, relatively well-tolerated, widely available, and inexpensive [97]. Clinicians use potassium bicarbonate less commonly, except in individuals requiring large amounts of a HCO_3_^−^ replacement (like in proximal renal tubular acidosis), which is associated with large potassium losses in response to treatment. Clinicians should avoid prescribing potassium bicarbonate in individuals with very low eGFRs (<25% of normal) because of the risk for potassium retention with hyperkalemia. Because citrate is metabolized to yield HCO_3_^−^, sodium citrate is often used in patients unable to tolerate NaHCO_3_. The use of sodium citrate is limited by its unpleasant taste and comparatively high expense and because it promotes gastric aluminum absorption [98]. Consequently, NaHCO_3_ is the more commonly used Na^+^-based alkali salt.

### 11.4. Remove Accumulated Acid: Acid-Binding Polymers

The non-absorbable polymer veverimer, still an investigational drug which is not available for clinical use, has been shown to increase serum [HCO_3_^−^] within 24 h of its administration [94] and to sustain a higher-than-placebo serum [HCO_3_^−^] for up to 52 weeks [99,100]. Although this sustained increase in serum [HCO_3_^−^] has been associated with both subjective and objective measures of improved physical function [99,100], it has not been associated with slowed CKD progression [101].

## 12. Conclusions

The continuum of disorders of H^+^ accumulation includes states in which accumulation is less than what is necessary to cause metabolic acidosis according to the serum acid–base parameters but is sufficient to cause organ injury, including to bones, muscles, and kidneys. These states of early-phase H^+^ stress appear most likely in individuals with early-stage CKD eating the highly H^+^-producing diets typical of modern society. Ongoing studies will determine clinically useful ways by which to identify early-phase H^+^ stress and, therefore, identify patients who are candidates for dietary H^+^ reduction, importantly including high proportions of F & Vs.
